# Neuroanatomical Changes in Leber’s Hereditary Optic Neuropathy: Clinical Application of 7T MRI Submillimeter Morphometry

**DOI:** 10.3390/brainsci10060359

**Published:** 2020-06-09

**Authors:** Kamil Jonak, Paweł Krukow, Mark Symms, Ryszard Maciejewski, Cezary Grochowski

**Affiliations:** 1 Department of Biomedical Engineering, Lublin University of Technology, 20-618 Lublin, Poland; 2 Department of Psychiatry, Psychotherapy and Early Intervention, Medical University of Lublin, 20-439 Lublin, Poland; 3 Department of Clinical Neuropsychiatry, Medical University of Lublin, 20-439 Lublin, Poland; pawelkrukow@umlub.pl; 4 GE Healthcare, Amersham Place, Amersham HP7 9NA, UK; MarkRoger.Symms@ge.com; 5 Department of Anatomy, Medical University of Lublin, 20-400 Lublin, Poland; maciejewski.r@gmail.com; 6 Laboratory of Virtual Man, Medical University of Lublin, 20-400 Lublin, Poland

**Keywords:** LHON, 7T MRI, morphometry, illness duration, mitochondrial

## Abstract

Leber’s hereditary optic neuropathy (LHON) is one of the mitochondrial diseases that causes loss of central vision, progressive impairment and subsequent degeneration of retinal ganglion cells (RGCs). In recent years, diffusion tensor imaging (DTI) studies have revealed structural abnormalities in visual white matter tracts, such as the optic tract, and optic radiation. However, it is still unclear if the disease alters only some parts of the white matter architecture or whether the changes also affect other subcortical areas of the brain. This study aimed to improve our understanding of morphometric changes in subcortical brain areas and their associations with the clinical picture in LHON by the application of a submillimeter surface-based analysis approach to the ultra-high-field 7T magnetic resonance imaging data. To meet these goals, fifteen LHON patients and fifteen age-matched healthy subjects were examined. For all individuals, quantitative analysis of the morphometric results was performed. Furthermore, morphometric characteristics which differentiated the groups were correlated with variables covering selected aspects of the LHON clinical picture. Compared to healthy controls (HC), LHON carriers showed significantly lower volume of both palladiums (left *p* = 0.023; right *p* = 0.018), the right accumbens area (*p* = 0.007) and the optic chiasm (*p* = 0.014). Additionally, LHON patients have significantly higher volume of both lateral ventricles (left *p* = 0.034; right *p* = 0.02), both temporal horns of the lateral ventricles (left *p* = 0.016; right *p* = 0.034), 3rd ventricle (*p* = 0.012) and 4th ventricle (*p* = 0.002). Correlation between volumetric results and clinical data showed that volume of both right and left lateral ventricles significantly and positively correlated with the duration of the illness (left *R* = 0.841, *p* = 0.002; right *R* = 0.755, *p* = 0.001) and the age of the LHON participants (left *R* = 0.656, *p* = 0.007; right *R* = 0.691, *p* = 0.004). The abnormalities in volume of the LHON patients’ subcortical structures indicate that the disease can cause changes not only in the white matter areas constituting visual tracts, but also in the other subcortical brain structures. Furthermore, the correlation between those results and the illness duration suggests that the disease might have a neurodegenerative nature; however, to fully confirm this observation, longitudinal studies should be conducted.

## 1. Introduction

Leber’s hereditary optic neuropathy (LHON) is one of the most frequent mitochondrial diseases of the optic nerve [1], characterized by maternally inherited genetic disorder, with young male predilection and loss of central vision, which is almost always bilateral and severe [2,3]. Although the disease was described for the first time in 1871 by the ophthalmologist Theodor Leber [4], it took researchers more than 100 years to identify several mutations in the mitochondrial DNA (mtDNA) that would affect mitochondrial oxidative phosphorylation [5,6]. Pathologically, changes in LHON show retinal ganglion cell degeneration with the axonal loss of the optic nerve and the thickening of the retinal nerve fiber layer (RNFL) [7]. These changes result in a decline in visual acuity (VA), permanent central scotoma and optic nerve atrophy.

Accelerated development of magnetic resonance imaging (MRI) methods allow researchers and clinicians to assess the neurodegenerative processes in many types of diseases associated with progressive blinding [8,9,10,11,12]. Anatomical changes observed in the LHON patients were consistent with the clinical picture of the disease. Studies have reported atrophy and increased T2-weighted signal intensity in the optic nerves [13,14], structural damage of the visual cortex and retinofugal pathway, which could be related to axonal degeneration secondary to the loss of retinal ganglion cells [15,16]. Recent studies with the application of diffusion transfer imagining (DTI) showed a significant decrease in fractional anisotropy (FA) in LHON patients [17] and revealed a microstructural alteration of the white matter (WM) [18].

Applications of the MRI method to measure neuronal atrophy are regarded as valid markers of disease state and progression [19]. However, variability in the shape and neuroanatomical configuration of individual brains may cause overlooking of structural differences by visual inspection or volume of interest (VOI) techniques. For more precise assessment of brain structure changes, voxel-based morphometry (VBM) and the surface-based (SB) approach have been applied to MRI analysis [20,21]. The VBM and SB methods are automated techniques that enable an assessment of the structure of the brain at the level of the voxel and appear to be well-established techniques for assessment of brain tissue loss in a wide spectrum of patients [22,23,24,25,26]. Morphometric studies with the application of VBM into Leber’s hereditary optic neuropathy showed reduction of the volume in the optic chiasm, optic tract, optic radiations and primary visual cortex [15]. Additionally, visual cortex changes were significantly correlated with global and temporal peripapillary retinal nerve fiber layer thickness [15]. Studies based on SB analysis in LHON revealed that the optic tract and the optic radiation differ from controls in radial and axial diffusivity [17].

Despite the mentioned MRI-based studies investigating LHON, it is still unclear how the disease, and its functional consequences associated with blindness, might influence individual subcortical structures, for example its volume. Previous researchers, who have confirmed the occurrence of gradual degeneration associated with prolonged duration of the disease, observed a unique process in which the RNFL changed from thickening in the acute stage to thinning in the subacute or chronic phase in LHON patients [27], as well as finding a significant correlation with the abnormalities within resting state brain networks [28]. Additionally, there is also a lack of publications describing detailed morphometric results from main subcortical brain structures such as basal ganglia or ventricles in the LHON patients. One of the reasons for this might be the fact that previous inquiries were based on 1.5 and 3T scanners giving less precise parcellation and segmentation results compared with 7T scanners [29,30]. Usage of 7T MRI machines enriches the images with spatial details not visible at lower field strengths due to the possibility of acquiring data with a higher resolution and Contrast to Noise Ratio (CNR), but without increasing the scan time [31]. High-field MRIs can also generate images with significantly improved contrast between deep brain grey matter tissue and WM [32]. Application of submillimeter voxel analysis to MRI volumetric study could substantially master the brain parcellation and segmentation effect and give more robust volumetric results [33]. Some studies have already provided evidence of improved identification of disease-specific morphological changes in the brains of patients with multiple sclerosis and Alzheimer’s disease using 7 T versus 3 T MRIs [34,35]. Ultra-high-field imaging also has enhanced sensitivity to detect fine-scale structural [36,37] and functional properties and changes in the brain [38]. Nevertheless, in accordance with our knowledge, there are no studies focused on morphological changes in the brains of LHON patients estimated with the application of ultra-high-field 7T MRI.

This study had two specific goals. First, we examined if there were any changes in subcortical structures associated with LHON disease. Previous studies clearly showed decreased volume of optic radiation and chiasm, changes in oxygen consumption [39], antioxidants [40], phosphorylation and mitochondrial ATP production [41] in LHON patients. Although, it is still unknown whether LHON mutations have a direct effect on subcortical brain structural properties. Second, our study is the first application of submillimeter high-field MRI for the evaluation of morphometric changes in LHON patients. The high precision of 7T MRI could reveal some significant associations between brain abnormalities and selected features of this disease’s clinical picture. Establishing such significant relationships might deepen the current knowledge regarding disease progression and its neuroanatomical basis. This applies first of all to the question regarding stability or progression of neuroanatomical changes accumulating with patients’ age and duration of illness.

## 2. Methods

### 2.1. Subjects

Initially, twenty-five patients with LHON were selected from a national health database. However, only 15 of them met the final inclusion criteria, which were the following: 11778G > A mitochondrial DNA mutation confirmed by genetic tests, participants couldn’t have known pathologies within the cerebrovascular system, were capable of signing informed consent, were over 18 years old, had at least 10 years of regular education and no familial history of severe neuropsychiatric disorders which would additionally affect the state of the nervous system. The patients did not suffer from hypertension, diabetes and any neurodegenerative or neurological diseases. Additionally, patients with any metallic implant, who were pregnant or breastfeeding, as well as those suffering from claustrophobia, were excluded from the study. Radiological assessment was carried out by an experienced neuroradiologist (25 years of experience) and a neuroanatomy specialist (40 years of experience). The healthy controls (HC) group was recruited from the local community after the clinical group was completed to guarantee the demographic matching of individuals from both samples. All of the participants were right-handed non-smokers with no history of chronic alcohol consumption. Blood pressure was measured in all participants and no abnormalities were found. All participants signed informed consent. This research was approved by the local medical ethics committee of the Medical University of Lublin (KE-0254/23/2017) and was carried out in compliance with national legislation and the Declaration of Helsinki. The scans were obtained at the Ecotech Complex, Lublin, Poland.

### 2.2. MRI Acquisition

Three-dimensional inversion recovery-prepared spoiled gradient echo (3D-SPGR “BRAVO”) was acquired from the 7T MRI with 32-channel coil at the Ecotech Complex, Lublin. Field of view was 220 × 220 × 180 mm and the acquisition matrix was 256 × 256 × 180. The images were reconstructed to a 512 × 512 matrix, giving a final voxel size of 0.43 × 0.43 × 1 mm. TE 2.6 ms, TR 6.6 ms, TI 450 ms, Flip angle 12 degrees, bandwidth +/− 31.25 kHz. Parallel imaging (ARC) factor 2 was used.

### 2.3. Image Analysis

The first step of analysis was the application of bias correction algorithms in SPM12 (http://www.fil.ion.ucl.ac.uk/spm; MATLAB R2018A version, Mathworks, Inc, Natick, MA, USA). Due to high field inhomogeneity in 7T MRI each structural volume was intensity bias corrected using the unified segmentation process [20]. Brain segmentation and volume calculations were performed using FreeSurfer (version 7.0) (https://surfer.nmr.mgh.harvard.edu). In this study we have not used the normal recon-all procedure but the submillimeter version of the recon-all procedure [33]. Nevertheless, the submillimeter recon-all procedure normal steps of data reconstructions, such as skull stripping, volumetric registration, normalization, volumetric labelling, segmentation, smoothing and cortical parcellation are also included (https://surfer.nmr.mgh.harvard.edu/fswiki/recon-all). As the submillimeter reconstruction was unstable with our native voxel size, we thus down-sampled voxel size into 0.5 mm^3^. During the pipeline procedure every participant’s brainmask was manually fixed by the radiologist and the surface inflation number was 100. After the initial preprocessing, quality assessment was conducted by the radiologist as well. Each slice was visually inspected for skull stripping errors, segmentation errors, normalization errors pial surface errors, and topological defects, following the FreeSurfer guidelines. The appropriate preprocessing steps were then repeated for the participants whose images required editing. After that, tabulated data of the segmented results were collected using two FreeSurfer summarizing scripts (i.e., aparcstats2table and asegstats2table).

### 2.4. Statistical Analysis

Due to the relatively small number of participants in each of the research groups, and the results of Shapiro-Wilk test showing non-compliance of our volumetric data with the normal distribution, groups were compared using the two-sided, non-parametric Mann-Whitney U-test for independent samples (*z*), with the statistical significance threshold set at *p* < 0.05. Each volumetric variable was individually compared between groups. The *χ^2^* test was applied to compare groups for qualitative variables, such as sex. After determining between-group differences regarding volumetric variables, these metrics, which significantly differentiated the groups, were included in the further step of the analysis. This consisted of establishing potential correlations between volumetrically altered structures and selected demographic and clinical variables in the LHON group, such as age and duration of illness. Correlation analysis regarding mentioned individual variables was carried out using the non-parametric Spearman R test. The level of statistical significance (ultimately *p* < 0.05) was corrected for the number of performed tests according to the false discovery rate (FDR) method.

## 3. Results

### 3.1. Participants

Table 1 presents demographic and clinical data on the studied groups. Samples did not differ significantly in terms of age (LHON = 36.21; HC = 32.53), sex (LHON = 86% male; HC = 66% male) or years of education (LHON = 15.33; HC = 16). In the LHON group, the duration of the illness lasted for about 10 years.

### 3.2. Volumetric Differences between Groups

**Figure 1:**
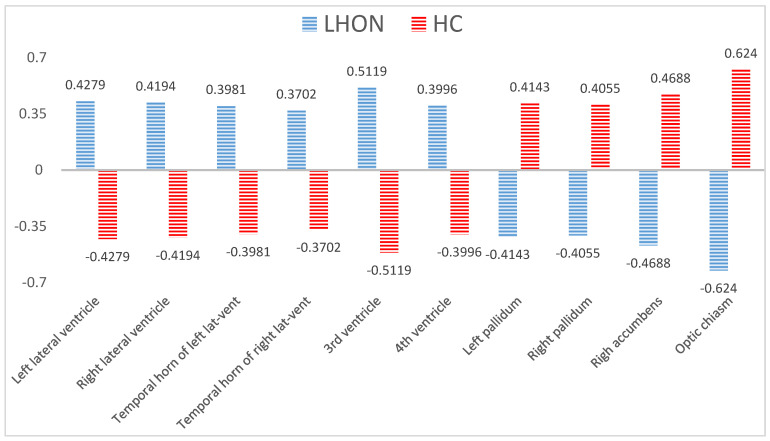
The figure displays a standardized volumetric data (z-scores) of ten neural structures significantly differing the groups according to non-parametric Mann-Whitney U-test. The raw volumetric indicators were standardized to cover results ranging from 96 to 10,246 mm3 and jointly show them in one line. The mean for both groups taken together was 0. Volumes of the 3rd ventricle and optic chiasm differentiated the groups for the most extent. The z-standardization of these result was made only for presentational purposes.

Table 2 presents results of volumetric analysis regarding HC and LHON groups. Statistical analysis revealed that compared to HC, LHON patients had significantly lower volume of both palladiums (left *p* = 0.023; right *p* = 0.018), right accumbens area (*p* = 0.007) and the optic chiasm (*p* = 0.014) (Figure 1). Additionally, LHON patients had significantly higher volume of both lateral ventricles (left *p* = 0.034; right *p* = 0.02), both temporal horns of lateral ventricles (left *p* = 0.016; right *p* = 0.034), 3rd ventricle (*p* = 0.012) and 4th ventricle (*p* = 0.002) (Figure 1).

### 3.3. Associations between Volumetric Results and Clinical Data of LHON Participants

**Figure 2:**
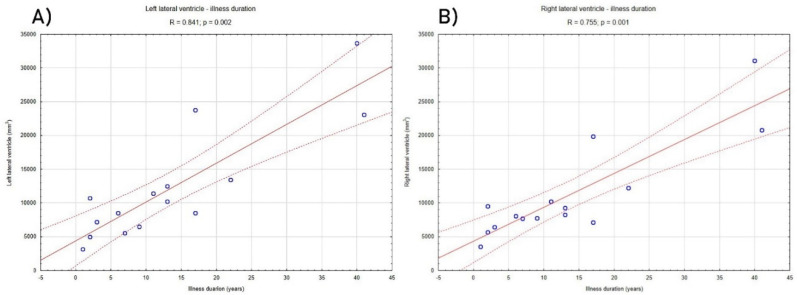
The figure shows correlation between duration of illness and the volume of left (**A**) and right (**B**) lateral ventricle in the LHON patients. *p*-value and *R* values were calculated using the non-parametric Spearman *R* test.

**Figure 3:**
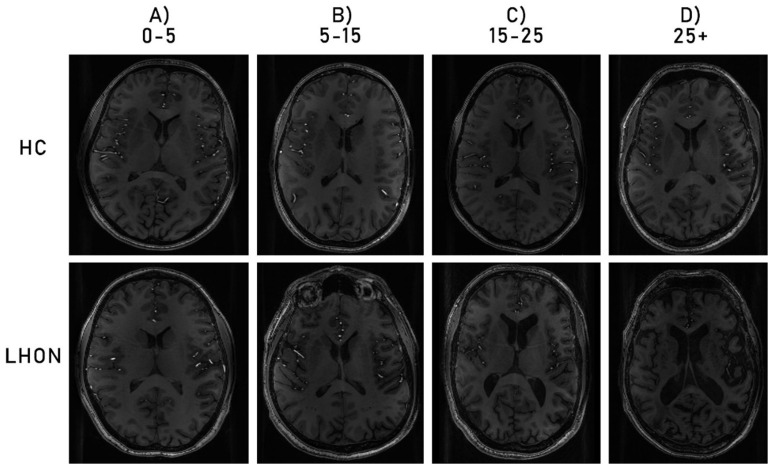
Comparison of T1 images from four LHON participants with different duration illness (**A**) 0–5 years; (**B**) 5–15 years; (**C**) 15–25 years; (**D**) 25+ years; with four HC participants of similar age and gender.

Analyses of relationships between volumetric data and clinical characteristics showed that volumes of both right and left lateral ventricles significantly and strongly correlated with the duration of the illness (left *R* = 0.841, *p* = 0.002; right *R* = 0.755, *p* = 0.001) (Figure 2) and age of participants from the LHON group (left *R* = 0.656, *p* = 0.007; right *R* = 0.691, *p* = 0.004). Figure 3 shows cross-sectional 7T MRI/T1 images of several LHON patients at four stages of disease duration (0–5, 5–15, 15–25, 25+; years) and age and sex-matched healthy controls. The qualitative comparison of these images suggests accelerated neural atrophy and enlargement of lateral ventricles in LHON patients compared with controls, which is in line with strong correlations between volume of ventricles and duration of illness in the LHON sample.

## 4. Discussion

In this study we showed the first application of submillimeter surface-based morphometry of ultra-high-field MRI data to investigate possible volumetric abnormalities of brain structures in LHON patients. Moreover, we have demonstrated that those neuronal changes were associated with selected features of the disease’s clinical picture. The most important findings were that compared to controls, LHON patients showed significantly decreased volume in a set of subcortical grey matter nuclei, including both palladiums, the right accumbens area and the optic chiasm. Additionally, LHON subjects have significantly higher volume of both lateral ventricles, both temporal horns of lateral ventricles, 3rd ventricle and 4th ventricle. All of these findings have met our major goal, which was to verify whether the brain abnormalities in LHON patients are limited only to brain areas constituting the visual system. Our main outcomes suggest that volumetric characteristics of selected subcortical structures not belonging to the visual system in LHON patients are also significantly altered. We have additionally documented significant correlations between some of the mentioned altered volume variables and clinical data in the LHON group. Correlations between volumetric results and clinical characteristics showed that the volume of both right and left lateral ventricles was significantly associated with the duration of the illness and the age of LHON participants. Observed results indicate that brain abnormalities in mitochondrial disease like LHON can progress with patients’ age and duration of illness.

### 4.1. Brain Morphological Abnormalities of the LHON Participants

Implementation of submillimeter surface-based morphometry into ultra-high-field MRI turned out to be robust tool for brain morphometric analysis in the LHON group. In the previous study, LHON patients had significant reduced volume of the bilateral primary visual cortex GM and reduced WM volume in the optic chiasm, optic tract—areas located in the optic radiations [15]. Reduction of optic chiasm volume, established also in our results, once again confirms how sensitive areas of the visual pathway are to the LHON disease. Compared with previous studies using 3T [42] and 1.5T MRIs [43], ultra-high-field 7T MRI also showed decreased volume of both pallidums and the right accumbens, which has not been reported earlier in LHON patients. Nevertheless, recent studies that also include Leber plus patients (LHON patients with dystonia) reported increased T2 signal intensity in the basal ganglia area; however, those cases are connected with a different mtDNA mutation: 10197G > A [44]. Furthermore, except for reduced volumetric parameters in LHON patients, we have also observed enlarged ventricular brain systems in this sample. Earlier investigations were focused on optic neuropathies and reported many WM abnormalities, specifically within the optic tract fibers [18]. Recent studies that combined DTI and quantitative T1 (qT1) measurements revealed volume loss along the optic radiation and suggested that the microstructural origins of the diffusivity changes can be different between the tracts, but the mechanisms of the myelin loss and abnormalities in diffusion parameters remain unclear [17]. In the current study we have not analyzed specific WM tracts, although several volumetric abnormalities in the brain ventricular systems of LHON patients were noticed. These findings could be a consequence of changes in several WM areas of the brain in LHON patients; hence, ventricular enlargement may be associated with progressive atrophy of different WM fibers in the brain.

### 4.2. Associations with Clinical Characteristics

Besides the mentioned morphometric anomalies, we have also revealed significant positive and strong correlations between the volume of ventricles and the LHON duration of the illness. Although there were also correlations between the same structures and the age of LHON participants, we have not found any significant correlations between age and ventricle volume in the controls group; therefore, it seems likely that progressive enlargement of ventricles was associated only with the age of LHON participants. Some previous studies confirmed that the duration of illness in the LHON group was correlated with the macular choroidal thickness [45]. Nevertheless, DTI studies focused on optic nerve quantitative assessment between chronic LHON patients and normal control subjects showed no correlation between illness duration and DTI parameters and suggest that damage along the visual pathways may occur relatively early from the disease’s clinical onset, or could be already present when the first symptoms are reported by the patient [46]. Ophthalmologic studies focused on the progressive changes in LHON patients showed structural changes in the ganglion cell layer and inner plexiform layer [47], as well as RNFL thickening [27]. Ventricle enlargement and increased CSF volume are reliable morphometric features of neural atrophy. These phenomena were observed in such typical neurodegenerative diseases as mild cognitive impairment, Alzheimer’s disease [48], Parkinson’s disease [49], Huntington’s disease [50] and in patients after traumatic brain injury (TBI) [51]. Considering the discussed findings, our study suggests, probably for the first time, that in LHON patients neurodegenerative changes might occur, and do not involve only those neural areas which are the major parts of the brain’s visual system.

### 4.3. Limitations

Some limitations must be considered when interpreting our results. Firstly, we have not used T2 data to improve pial surfaces during the segmentation protocol in Freesurfer software; however, pial surface errors were manually examined and corrected by a neurologist during the analysis. Second, our final sample size was limited to only fifteen patients, which might at least partially reduce the statistical power of the main findings. There is no doubt that future studies need to include a larger sample in order to confirm our findings. Third, it would be beneficial to directly compare LHON-related morphometric results taken from 1.5T, 3T and 7T MRI scanners to precisely verify the assumption that ultra-high-field morphometry originating from the 7T scanner brings substantially improved data compared with those obtained from 1.5T or 3T scanners. Furthermore, to analyze the relationship between the volume loss of subcortical structures and the atrophy of the white matter fibers that form the visual pathway, a hybrid study that combines submillimeter morphometry and diffusion imaging should be done in an LHON cohort. Additionally, we did not conduct an additional examination of ophthalmic parameters of our patients, due to the fact that according to information taken from them in the initial interview, patients described themselves as functionally blind. The statistical analysis regarding group differences of the volumetric indicators was conducted with a relatively simple, non-parametric test, which did not control for potential confounds such as demographic characteristics. However, as stated earlier, participants included in the final research groups were very alike in this respect, which, in our opinion, justified application of a direct variable comparison with a non-parametric test.

## 5. Conclusions

In conclusion, this is the first study that utilized ultra-high field MRI data for quantification of volumetric, subcortical brain changes in a representative population of LHON patients. Comparative analysis between controls and LHON patients revealed decreased volume in subcortical brain structures: both pallidums and the right accumbens area. Additionally, increased volume of brain ventricular systems occurred. The observed enlargement of both right and left lateral ventricles significantly and strongly correlated with the duration of the illness, which is of special interest, considering that this is the first study with such an observation in this particular group of patients. Nevertheless, future longitudinal quantitative MRI and in vitro studies are necessary to track the progression of the detected morphological changes in Leber’s disease.

## Figures and Tables

**Table 1:** Demographic and clinical data of research groups.

	LHON (*n* = 15) M (SD)	HC (*n* = 15) M (SD)	*z* Value or *χ2*	*p*
Age (years)	36.21 (14.41)	32.53 (7.42)	0.024	0.981
Education (years)	15.33 (1.98)	16 (1.55)	−1.823	0.674
Sex (% male)	86	66	1.671	0.192
Duration of illness (months)	132 (144.32)			
Education (years)	15.33 (1.98)	16 (1.55)	−1.823	0.674
Sex (% male)	86	66	1.671	0.192
Duration of illness (months)	132 (144.32)			
Mitochondrial mutation 11778G > A (%)	100			

**Table 2:** Results of volumetric analysis (mm3).

	LHON (*N* = 15)	HC (*N* = 15)		
	M	M	*Z*	*p*
Left lateral ventricle	10,246	6646	2.115	0.035 *
Temporal horn of left lat-vent	567	289	2.406	0.016 *
Left Cerebellum WM	20,581	19,375	0.249	0.804
Left Cerebellum Cortex	52,479	53,110	0.415	0.678
Left Thalamus	7507	7205	−0.249	0.804
Left Caudate	3786	3942	−0.124	0.901
Left Putamen	5616	5262	0.539	0.590
Left Pallidum	1509	1727	−2.281	0.023 *
3rd Ventricle	1628	1314	2.489	0.013 *
4th Ventricle	2931	1996	3.069	0.002 *
Left Hippocampus	3944	3989	0.249	0.804
Left Amygdala	1364	1412	−0.332	0.740
Cerebrospinal fluid	1789	1488	0.747	0.455
Left Accumbens area	553	499	0.664	0.507
Left Choroid plexus	384	335	0.830	0.407
Right lateral ventricle	8276	6179	2.323	0.020 *
Temporal horn of right lat-vent	414	305	2.115	0.034 *
Right Cerebellum WM	1,9727	1,7227	1.078	0.281
Right Cerebellum Cortex	5,2944	5,1682	0.249	0.804
Right Thalamus	7119	7378	−0.041	0.967
Right Caudate	4064	3864	0.995	0.320
Right Putamen	5875	5542	0.788	0.431
Right Pallidum	1236	1531	−2.364	0.018 *
Right Hippocampus	3992	3761	1.908	0.056
Right Amygdala	1666	1666	0.041	0.967
Right Accumbens area	555	711	−2.696	0.007 *
Right Choroid plexus	414	381	0.664	0.508
Optic Chiasm	96	158	−2.530	0.011 *
CC Posterior	573	671	−0.456	0.648
CC Mid Posterior	548	477	−0.166	0.868
CC Central	506	476	−0.166	0.868
CC Mid Anterior	524	520	−0.083	0.934
CC Anterior	921	823	0.290	0.772
Left Cortex	251,888	248,114	0.456	0.648
Right Cortex	255,895	244,263	0.539	0.590
Cortex	506,176	496,939	0.581	0.561
Left Cerebral WM	228,307	239,037	−0.373	0.709
Right Cerebral WM	238,648	248,453	−0.083	0.934
WM	459,714	487,491	−0.332	0.740
Sub-cortical GM	57,462	57,970	−0.166	0.868
Total GM	670,972	664,681	0.456	0.648

***Note.*** CC—Corpus callosum, WM—White matter, GM—Gray matter, lat-vent—Lateral ventricle, M—Median, SD—Standard derivation, Z—Mann-Whitney test, *—significant difference between groups.
